# Supplementary material for: The role of PDF neurons in setting the preferred temperature before dawn in Drosophila
Source: eLife. 2017 May 2;6:e23206. doi: 10.7554/eLife.23206 (PMC5449184; doi:10.7554/eLife.23206)
Supplement: Supplementary file 1. — The preferred temperatures among Gal4/UAS, Gal4/+ and UAS/+ flies were analyzed using One-way ANOVA and Tukey-Kramer tests. In each time zone, F and p values and degrees of freedom are shown. The comparison between Gal4/UAS and Gal4/+, Gal4/UAS and UAS/+ as well as Gal4/+ and UAS/+ are shown in green, blue and red, respectively (****p<0.0001, ***p<0.001, **p<0.01 or *p<0.05). Stars are shown in Figures 1 and 4D–F and Figure 4—figure supplement 1C–D when Gal4/UAS are statistically different from both Gal4/+ (stars in green) and UAS/+ (stars in blue). These time zones are highlighted in orange. DOI: http://dx.doi.org/10.7554/eLife.23206.017 [file elife-23206-supp1.docx]

|  | |  |  |  |  |
| --- | --- | --- | --- | --- | --- |
| **Figure 1A:Pdf-Gal4/UAS-Kir** | | | **Tukey-kramer test** | | |
|  | **one-way ANOVA** | **F (DFn, DFd)** | **Gal4/UAS vs Gal4/+** | **Gal4/UAS vs UAS/+** | **Gal4/+ vs UAS/+** |
| **ZT1-3** | **P = 0.0087** | **F (2, 19) = 6.160** | ****** | **ns** | ***** |
| **ZT4-6** | **P = 0.0073** | **F (2, 14) = 7.144** | ***** | **ns** | ***** |
| **ZT7-9** | **P = 0.1270** | **F (2, 10) =2.555** | **ns** | **ns** | **ns** |
| **ZT10-12** | **P = 0.1562** | **F (2, 14) = 2.126** | **ns** | **ns** | **ns** |
| **ZT13-15** | **P = 0.7267** | **F (2, 21) = 0.3242** | **ns** | **ns** | **ns** |
| **ZT16-18** | **P = 0.0353** | **F (2, 22) = 3.907** | ***** | **ns** | **ns** |
| **ZT19-21** | **P = 0.0008** | **F (2, 15) = 15.88** | ******* | ***** | ***** |
| **ZT22-24** | **P < 0.0001** | **F (2, 24) = 33.48** | ******* | ****** | ******* |
|  |  |  |  |  |  |
| **Figure 1B:Pdf-Gal4/UAS-Clock delta** | | | **Tukey-kramer test** | | |
|  | **one-way ANOVA** | **F (DFn, DFd)** | **Gal4/UAS vs Gal4/+** | **Gal4/UAS vs UAS/+** | **Gal4/+ vs UAS/+** |
| **ZT1-3** | **P = 0.0372** | **F (2, 16) = 4.070** | **ns** | **ns** | **ns** |
| **ZT4-6** | **P = 0.0081** | **F (2, 18) = 6.370** | **ns** | ****** | **ns** |
| **ZT7-9** | **P = 0.0307** | **F (2, 14) = 4.514** | **ns** | ***** | **ns** |
| **ZT10-12** | **P = 0.2474** | **F (2, 15) = 1.535** | **ns** | **ns** | **ns** |
| **ZT13-15** | **P = 0.8742** | **F (2, 27) = 0.1351** | **ns** | **ns** | **ns** |
| **ZT16-18** | **P = 0.0071** | **F (2, 22) = 6.242** | ****** | **ns** | **ns** |
| **ZT19-21** | **P < 0.0001** | **F (2, 18) = 25.60** | ******* | ****** | ***** |
| **ZT22-24** | **P < 0.0001** | **F (2, 15) = 22.60** | ******* | ******* | **ns** |
|  |  |  |  |  |  |
| **Figure 4D:TrpA1-SH-Gal4/UAS-Kir** | | | **Tukey-kramer test** | | |
|  | **one-way ANOVA** | **F (DFn, DFd)** | **Gal4/UAS vs Gal4/+** | **Gal4/UAS vs UAS/+** | **Gal4/+ vs UAS/+** |
| **ZT1-3** | **P = 0.0044** | **F (2, 23) = 6.948** | ****** | ****** | **ns** |
| **ZT4-6** | **P = 0.0304** | **F (2, 15) = 4.447** | ***** | **ns** | **ns** |
| **ZT7-9** | **P = 0.9811** | **F (2, 21) = 0.01910** | **ns** | **ns** | **ns** |
| **ZT10-12** | **P = 0.4599** | **F (2, 18) = 0.8112** | **ns** | **ns** | **ns** |
| **ZT13-15** | **P = 0.0820** | **F (2, 26) = 2.758** | **ns** | **ns** | **ns** |
| **ZT16-18** | **P = 0.0457** | **F (2, 24) = 3.518** | ***** | **ns** | **ns** |
| **ZT19-21** | **P = 0.0018** | **F (2, 22) = 8.538** | ****** | ***** | **ns** |
| **ZT22-24** | **P < 0.0001** | **F (2, 39) = 15.54** | ******** | ****** | **ns** |
|  |  |  |  |  |  |
| **Figure 4E:Pdf-Gal4/UAS-5HT1B-RNAi** | | | **Tukey-kramer test** | | |
|  | **one-way ANOVA** | **F (DFn, DFd)** | **Gal4/UAS vs Gal4/+** | **Gal4/UAS vs UAS/+** | **Gal4/+ vs UAS/+** |
| **ZT1-3** | **P = 0.0866** | **F (2, 23) = 2.727** | **ns** | **ns** | **ns** |
| **ZT4-6** | **P = 0.5715** | **F (2, 23) = 0.5734** | **ns** | **ns** | **ns** |
| **ZT7-9** | **P = 0.0355** | **F (2, 16) = 4.141** | **ns** | ***** | **ns** |
| **ZT10-12** | **P = 0.2615** | **F (2, 15) = 1.469** | **ns** | **ns** | **ns** |
| **ZT13-15** | **P = 0.0901** | **F (2, 28) = 2.626** | **ns** | **ns** | **ns** |
| **ZT16-18** | **P = 0.0086** | **F (2, 28) = 5.670** | ***** | ****** | **ns** |
| **ZT19-21** | **P = 0.1386** | **F (2, 25) = 2.141** | **ns** | **ns** | **ns** |
| **ZT22-24** | **P = 0.0001** | **F (2, 24) = 13.28** | ******* | ******* | **ns** |
|  |  |  |  |  |  |
| **Figure 4F:R6-Gal4/UAS-5HT1B-RNAi** | | | **Tukey-kramer test** | | |
|  | **one-way ANOVA** | **F (DFn, DFd)** | **Gal4/UAS vs Gal4/+** | **Gal4/UAS vs UAS/+** | **Gal4/+ vs UAS/+** |
| **ZT1-3** | **P = 0.2732** | **F (2, 23) = 1.373** | **ns** | **ns** | **ns** |
| **ZT4-6** | **P = 0.6345** | **F (2, 20) = 0.4655** | **ns** | **ns** | **ns** |
| **ZT7-9** | **P = 0.6443** | **F (2, 15) = 0.4528** | **ns** | **ns** | **ns** |
| **ZT10-12** | **P = 0.5230** | **F (2, 16) = 0.6752** | **ns** | **ns** | **ns** |
| **ZT13-15** | **P = 0.0160** | **F (2, 26) = 4.870** | **ns** | ***** | **ns** |
| **ZT16-18** | **P = 0.2276** | **F (2, 23) = 1.580** | **ns** | **ns** | **ns** |
| **ZT19-21** | **P = 0.0002** | **F (2, 29) = 11.25** | ****** | ******* | **ns** |
| **ZT22-24** | **P = 0.0001** | **F (2, 25) = 12.90** | ******* | ******* | **ns** |
|  |  |  |  |  |  |
| **Figure 4 Suppl Fig. 1C:TrpA1-SH-Gal4/**  **UAS-TrpA1-RNAI** | | | **Tukey-kramer test** | | |
|  | **one-way ANOVA** | **F (DFn, DFd)** | **Gal4/UAS vs Gal4/+** | **Gal4/UAS vs UAS/+** | **Gal4/+ vs UAS/+** |
| **ZT1-3** | **P = 0.0018** | **F (2, 16) = 9.663** | ***** | **ns** | ****** |
| **ZT4-6** | **P = 0.0399** | **F (2, 18) = 3.873** | **ns** | **ns** | ***** |
| **ZT7-9** | **P = 0.6195** | **F (2, 15) = 0.4945** | **ns** | **ns** | **ns** |
| **ZT10-12** | **P = 0.9972** | **F (2, 15) = 0.0028** | **ns** | **ns** | **ns** |
| **ZT13-15** | **P = 0.3236** | **F (2, 22) = 1.188** | **ns** | **ns** | **ns** |
| **ZT16-18** | **P = 0.7229** | **F (2, 19) = 0.3302** | **ns** | **ns** | **ns** |
| **ZT19-21** | **P = 0.0221** | **F (2, 18) = 4.749** | ***** | **ns** | **ns** |
| **ZT22-24** | **P = 0.0206** | **F (2, 28) = 4.472** | ***** | ***** | **ns** |
|  |  |  |  |  |  |
| **Figure 4 Suppl Fig. 1D:NP0002-Gal4/**  **UAS-TrpA1-RNAI** | | | **Tukey-kramer test** | | |
|  | **one-way ANOVA** | **F (DFn, DFd)** | **Gal4/UAS vs Gal4/+** | **Gal4/UAS vs UAS/+** | **Gal4/+ vs UAS/+** |
| **ZT1-3** | **P = 0.0764** | **F (2, 15) = 3.068** | **ns** | **ns** | **ns** |
| **ZT4-6** | **P = 0.3902** | **F (2, 15) = 1.003** | **ns** | **ns** | **ns** |
| **ZT7-9** | **P = 0.3160** | **F (2, 15) = 1.245** | **ns** | **ns** | **ns** |
| **ZT10-12** | **P = 0.7305** | **F (2, 12) = 0.3223** | **ns** | **ns** | **ns** |
| **ZT13-15** | **P = 0.4422** | **F (2, 20) = 0.8501** | **ns** | **ns** | **ns** |
| **ZT16-18** | **P = 0.0862** | **F (2, 16) = 2.868** | **ns** | **ns** | **ns** |
| **ZT19-21** | **P = 0.1714** | **F (2, 14) = 2.006** | **ns** | **ns** | **ns** |
| **ZT22-24** | **P = 0.0176** | **F (2, 20) = 4.976** | ***** | ***** | **ns** |
