# Supplementary material for: The role of PDF neurons in setting the preferred temperature before dawn in Drosophila
Source: eLife. 2017 May 2;6:e23206. doi: 10.7554/eLife.23206 (PMC5449184; doi:10.7554/eLife.23206)
Supplement: Supplementary file 2. — The preferred temperatures among Pdf-Gal4/+, UAS-Kir/+, UAS-∆clock/+, UAS-5-HT1B-RNAi/+ and R6-Gal4 flies were analyzed using one-way ANOVA and Tukey-Kramer tests. In the each time zone, F and p values and degrees of freedom are shown. ***p<0.001, **p<0.01 or *p<0.05. DOI: http://dx.doi.org/10.7554/eLife.23206.018 [file elife-23206-supp2.docx]

|  |  |  |  |  |  |
| --- | --- | --- | --- | --- | --- |
| **ZT1-3** |  |  |  |  |  |
| **ANOVA p = 0.0361, F (4, 36)=2.882** | **Pdf-Gal4/+** | **UAS-KIR/+** | **UAS-delta-clock/+** | **UAS-5-HT1B-RNAI/+** | **R6-Gal4/+** |
| **Pdf-Gal4/+** |  | **ns** | **ns** | **ns** | **ns** |
| **UAS-KIR/+** |  |  | **ns** | **ns** | **ns** |
| **UAS-delta-clock/+** |  |  |  | **ns** | **ns** |
| **UAS-5-HT1B-RNAI/+** |  |  |  |  | **ns** |
| **R6-Gal4/+** |  |  |  |  |  |
|  |  |  |  |  |  |
| **ZT4-6** |  |  |  |  |  |
| **ANOVA p = 0.0071, F (4, 29)=4.339** | **Pdf-Gal4/+** | **UAS-KIR/+** | **UAS-delta-clock/+** | **UAS-5-HT1B-RNAI/+** | **R6-Gal4/+** |
| **Pdf-Gal4/+** |  | **ns** | **ns** | **ns** | **ns** |
| **UAS-KIR/+** |  |  | ****** | **ns** | **ns** |
| **UAS-delta-clock/+** |  |  |  | **ns** | ***** |
| **UAS-5-HT1B-RNAI/+** |  |  |  |  | **ns** |
| **R6-Gal4/+** |  |  |  |  |  |
|  |  |  |  |  |  |
| **ZT7-9** |  |  |  |  |  |
| **ANOVA p = 0.0504, F (4, 23)=2.788** | **Pdf-Gal4/+** | **UAS-KIR/+** | **UAS-delta-clock/+** | **UAS-5-HT1B-RNAI/+** | **R6-Gal4/+** |
| **Pdf-Gal4/+** |  | **ns** | **ns** | **ns** | **ns** |
| **UAS-KIR/+** |  |  | **ns** | **ns** | **ns** |
| **UAS-delta-clock/+** |  |  |  | **ns** | **ns** |
| **UAS-5-HT1B-RNAI/+** |  |  |  |  | **ns** |
| **R6-Gal4/+** |  |  |  |  |  |
|  |  |  |  |  |  |
| **ZT10-12** |  |  |  |  |  |
| **ANOVA p = 0.2115, F (4, 26)=1.572** | **Pdf-Gal4/+** | **UAS-KIR/+** | **UAS-delta-clock/+** | **UAS-5-HT1B-RNAI/+** | **R6-Gal4/+** |
| **Pdf-Gal4/+** |  | **ns** | **ns** | **ns** | **ns** |
| **UAS-KIR/+** |  |  | **ns** | **ns** | **ns** |
| **UAS-delta-clock/+** |  |  |  | **ns** | **ns** |
| **UAS-5-HT1B-RNAI/+** |  |  |  |  | **ns** |
| **R6-Gal4/+** |  |  |  |  |  |
|  |  |  |  |  |  |
| **ZT13-15** |  |  |  |  |  |
| **ANOVA p = 0.0434, F (4, 35)=2.751** | **Pdf-Gal4/+** | **UAS-KIR/+** | **UAS-delta-clock/+** | **UAS-5-HT1B-RNAI/+** | **R6-Gal4/+** |
| **Pdf-Gal4/+** |  | **ns** | **ns** | **ns** | **ns** |
| **UAS-KIR/+** |  |  | **ns** | **ns** | **ns** |
| **UAS-delta-clock/+** |  |  |  | **ns** | **ns** |
| **UAS-5-HT1B-RNAI/+** |  |  |  |  | **ns** |
| **R6-Gal4/+** |  |  |  |  |  |
|  |  |  |  |  |  |
| **ZT16-18** |  |  |  |  |  |
| **ANOVA p = 0.1795, F (4, 36)=1.665** | **Pdf-Gal4/+** | **UAS-KIR/+** | **UAS-delta-clock/+** | **UAS-5-HT1B-RNAI/+** | **R6-Gal4/+** |
| **Pdf-Gal4/+** |  | **ns** | **ns** | **ns** | **ns** |
| **UAS-KIR/+** |  |  | **ns** | **ns** | **ns** |
| **UAS-delta-clock/+** |  |  |  | **ns** | **ns** |
| **UAS-5-HT1B-RNAI/+** |  |  |  |  | **ns** |
| **R6-Gal4/+** |  |  |  |  |  |
|  |  |  |  |  |  |
| **ZT19-21** |  |  |  |  |  |
| **ANOVA p = 0.0221, F (4, 40)=3.219** | **Pdf-Gal4/+** | **UAS-KIR/+** | **UAS-delta-clock/+** | **UAS-5-HT1B-RNAI/+** | **R6-Gal4/+** |
| **Pdf-Gal4/+** |  | **ns** | **ns** | **ns** | **ns** |
| **UAS-KIR/+** |  |  | **ns** | **ns** | **ns** |
| **UAS-delta-clock/+** |  |  |  | **ns** | **ns** |
| **UAS-5-HT1B-RNAI/+** |  |  |  |  | **ns** |
| **R6-Gal4/+** |  |  |  |  |  |
|  |  |  |  |  |  |
| **ZT22-24** |  |  |  |  |  |
| **ANOVA p < 0.0001, F (4, 42)=9.356** | **Pdf-Gal4/+** | **UAS-KIR/+** | **UAS-delta-clock/+** | **UAS-5-HT1B-RNAI/+** | **R6-Gal4/+** |
| **Pdf-Gal4/+** |  | ******* | **ns** | **ns** | **ns** |
| **UAS-KIR/+** |  |  | ***** | ******* | ******* |
| **UAS-delta-clock/+** |  |  |  | **ns** | **ns** |
| **UAS-5-HT1B-RNAI/+** |  |  |  |  | **ns** |
| **R6-Gal4/+** |  |  |  |  |  |
