# Supplementary material for: The role of PDF neurons in setting the preferred temperature before dawn in Drosophila
Source: eLife. 2017 May 2;6:e23206. doi: 10.7554/eLife.23206 (PMC5449184; doi:10.7554/eLife.23206)
Supplement: Supplementary file 3. — The daytime TPR of each fly line was analyzed using one-way ANOVA. The preferred temperatures of ZT1-3 were further compared to that of each time zone (ZT 4–6, 7–9 or 10–12) by Tukey-Kramer tests. F and p values and degrees of freedom are shown. ***p<0.001, **p<0.01 or *p<0.05 DOI: http://dx.doi.org/10.7554/eLife.23206.019 [file elife-23206-supp3.docx]

|  | |  |  |  |  |  |  |  |  |  | |  |  |  |  |  |  |  |  |
| --- | --- | --- | --- | --- | --- | --- | --- | --- | --- | --- | --- | --- | --- | --- | --- | --- | --- | --- | --- |
| ***pdfG4 x UAS-kir*** | |  |  | ***pdfG4 x UAS-Δclk*** | | |  | ***pdfG4 x UAS-5HT1B-RNAi*** | | | | | ***R6G4 x UAS-5HT1B-RNAi*** | | | | ***TrpA1G4 x UAS-kir*** | | |
| Comparison (ZT) | | P value |  | Comparison (ZT) | | P value |  | Comparison (ZT) | | P value | |  | Comparison (ZT) | | P value |  | Comparison (ZT) | | P value |
|  | 4-6 | ns |  |  | 4-6 | ns |  |  | 4-6 | ns | |  |  | 4-6 | ns |  |  | 4-6 | ns |
| 1-3 | 7-9 | ns |  | 1-3 | 7-9 | ns |  | 1-3 | 7-9 | ns | |  | 1-3 | 7-9 | ns |  | 1-3 | 7-9 | ns |
|  | 10-12 | ✽ |  |  | 10-12 | ✽✽ |  |  | 10-12 | ns | |  |  | 10-12 | ✽ |  |  | 10-12 | ns |
| 4-6 | 7-9 | ns |  | 4-6 | 7-9 | ns |  | 4-6 | 7-9 | ns | |  | 4-6 | 7-9 | ns |  | 4-6 | 7-9 | ns |
|  | 10-12 | ns |  |  | 10-12 | ns |  |  | 10-12 | ns | |  |  | 10-12 | ns |  |  | 10-12 | ns |
| 7-9 | 10-12 | ns |  | 7-9 | 10-12 | ns |  | 7-9 | 10-12 | ns | |  | 7-9 | 10-12 | ns |  | 7-9 | 10-12 | ns |
| **One way ANOVA p=0.0090** | | |  | **One way ANOVA p=0.0088** | | |  | **One way ANOVA p= 0.2169** | | | | | **One way ANOVA p= 0.0452** | | | | **One way ANOVA p=0.4756** | | |
| **F(3, 20)= 5.066** | |  |  | **F(3, 20)= 5.093** | |  |  | **F(3, 31)= 1.568** | |  | |  | **F(3, 20)= 3.206** | |  |  | **F(3, 34)= 0.8514** | |  |
|  |  |  |  |  |  |  |  |  |  |  | |  |  |  |  |  |  |  |  |
| ***pdfG4 x w1118*** | |  |  | ***UAS-kir x w1118*** | | |  | ***UAS-Δclk x w1118*** | | | |  | ***UAS-5HT1B-RNAi x w1118*** | | | | ***TrpA1G4 x w1118*** | | |
| Comparison (ZT) | | P value |  | Comparison (ZT) | | P value |  | Comparison (ZT) | | P value | |  | Comparison (ZT) | | P value |  | Comparison (ZT) | | P value |
|  | 4-6 | ns |  |  | 4-6 | ns |  |  | 4-6 | ns | |  |  | 4-6 | ns |  |  | 4-6 | ns |
| 1-3 | 7-9 | ns |  | 1-3 | 7-9 | ns |  | 1-3 | 7-9 | ns | |  | 1-3 | 7-9 | ✽✽ |  | 1-3 | 7-9 | ✽ |
|  | 10-12 | ns |  |  | 10-12 | ✽ |  |  | 10-12 | ns | |  |  | 10-12 | ✽✽ |  |  | 10-12 | ✽✽✽ |
| 4-6 | 7-9 | ns |  | 4-6 | 7-9 | ns |  | 4-6 | 7-9 | ns | |  | 4-6 | 7-9 | ns |  | 4-6 | 7-9 | ns |
|  | 10-12 | ✽ |  |  | 10-12 | ns |  |  | 10-12 | ns | |  |  | 10-12 | ns |  |  | 10-12 | ✽✽ |
| 7-9 | 10-12 | ns |  | 7-9 | 10-12 | ns |  | 7-9 | 10-12 | ns | |  | 7-9 | 10-12 | ns |  | 7-9 | 10-12 | ns |
| **One way ANOVA p= 0.0206** | | | | **One way ANOVA p=0.0166** | | |  | **One way ANOVA p= 0.1183** | | | | | **One way ANOVA p= 0.0012** | | | | **One way ANOVA p<0.0001** | | |
| **F(3, 20)= 4.079** | |  |  | **F(3, 17)= 4.519** | |  |  | **F(3, 23)= 2.176** | |  | |  | **F(3, 26)= 7.121** | |  |  | **F(3, 26)= 11.25** | |  |
|  |  |  |  |  |  |  |  |  |  |  | |  |  |  |  |  |  |  |  |
| ***NP0002G4 x***  ***UAS-TrpA1-RNAi*** | | |  | ***R6G4 x w1118*** | |  |  | ***UAS-TrpA1-RNAi x w1118*** | | | | | ***NP0002G4 x w1118*** | | |  | ***TrpA1G4 x***  ***UAS-TrpA1-RNAi*** | | |
| Comparison (ZT) | | P value |  | Comparison (ZT) | | P value |  | Comparison (ZT) | | P value | |  | Comparison (ZT) | | P value |  | Comparison (ZT) | | P value |
|  | 4-6 | ns |  |  | 4-6 | ns |  |  | 4-6 | ns | |  |  | 4-6 | ns |  |  | 4-6 | ns |
| 1-3 | 7-9 | ns |  | 1-3 | 7-9 | ns |  | 1-3 | 7-9 | ns | |  | 1-3 | 7-9 | ns |  | 1-3 | 7-9 | ns |
|  | 10-12 | ns |  |  | 10-12 | ✽ |  |  | 10-12 | ns | |  |  | 10-12 | ✽✽✽ |  |  | 10-12 | ns |
| 4-6 | 7-9 | ns |  | 4-6 | 7-9 | ns |  | 4-6 | 7-9 | ns | |  | 4-6 | 7-9 | ns |  | 4-6 | 7-9 | ns |
|  | 10-12 | ns |  |  | 10-12 | ✽ |  |  | 10-12 | ns | |  |  | 10-12 | ✽✽ |  |  | 10-12 | ✽ |
| 7-9 | 10-12 | ns |  | 7-9 | 10-12 | ns |  | 7-9 | 10-12 | ns | |  | 7-9 | 10-12 | ✽✽ |  | 7-9 | 10-12 | ns |
| **One way ANOVA p= 0.2505** | | | | **One way ANOVA p=0.0131** | | |  | **One way ANOVA p= 0.9394** | | | | | **One way ANOVA p= 0.0004** | | | | **One way ANOVA p=0.0353** | | |
| **F(3, 21)= 1.473** | |  |  | **F(3, 28)= 4.284** | |  |  | **F(3, 21)= 0.1329** | | |  |  | **F(3, 15)= 11.24** | |  |  | **F(3, 21)= 3.600** | |  |
